# Attention-Enhanced Feature-Based Point Cloud Completion Network for Precision Parts

**DOI:** 10.3390/s26134236

**Published:** 2026-07-03

**Authors:** Hongfei Zu, Chenzan Wang, Xuwen Chen, Ke Zheng, Enhao Li, Zhangwei Chen

**Affiliations:** 1 School of Mechanical Engineering, Zhejiang Sci-Tech University, Hangzhou 310018, China; 2 Zhejiang Premax Technology Co., Ltd., Ningbo 315048, China; 3 State Key Laboratory of Fluid Power and Mechatronic Systems, Zhejiang University, Hangzhou 310058, China

**Keywords:** point cloud completion, 3D scanning, attention mechanism, precision parts

## Abstract

When acquiring point cloud data of precision parts using 3D scanning devices, occlusion or equipment limitations often lead to sparse and incomplete data, resulting in the distortion or loss of key geometric features. To address this issue, this study proposes an attention-enhanced feature-based point cloud completion network for precision parts, using precision bearing rings as an example to construct a dedicated completion dataset for training. The proposed network adopts an encoder–decoder architecture. In the encoder stage, a curvature-weighted sampling feature extraction module and spatial attention mechanism are introduced to extract both local and global features from the incomplete point cloud, followed by multilevel feature fusion. The multiscale features extracted by the encoder are then fed into the decoder, which hierarchically and progressively predicts the missing regions of the point cloud. Finally, an adversarial generation module incorporating a biased attention mechanism enhances the sensitivity of the network to geometric structural differences, thereby producing a complete and refined point cloud as the final output. Experimental results show that on the ShapeNet-part dataset, the proposed network achieves average CD, Pred → GT, and GT → Pred errors of 4.663, 2.459, and 2.457, respectively, representing reductions of 10.8%, 4.7%, and 8.1%, respectively, compared with the mainstream PF-Net completion network. On the bearing ring dataset constructed in this study, the average CD, Pred → GT, and GT → Pred errors were 0.497, 1.064, and 0.601, respectively, decreasing by 9.3%, 16.3%, and 16.2%, respectively, relative to PF-Net. Moreover, the proposed network effectively completed the point clouds of various missing parts, demonstrating its robustness across different types of precision parts.

## 1. Introduction

A 3D point cloud is a collection of spatial coordinate points within a three-dimensional coordinate system that exhibits disorder and lacks connectivity. Unlike polygonal mesh surfaces, point clouds do not require the storage or maintenance of mesh connectivity or topological consistency. Owing to their simplicity, flexibility, and powerful representational capabilities, point clouds have been extensively applied in numerous research fields such as autonomous driving, non-contact inspection, and robotics [1,2,3]. In the field of precision inspection, point cloud data are of significant importance and research value. Through 3D imaging technologies such as laser scanners and optical interferometers, point cloud data enables non-contact, high-precision capture of surface geometric features on precision parts. This effectively addresses the low efficiency and risk of scratching workpieces associated with the traditional contact-based inspection methods.

Taking bearing ring inspection as an example, during actual inspection processes, data sparsity or incompleteness frequently occurs because of self-occlusion and external occlusion of the workpiece, limitations in the measurement instrument resolution, and other human factors. This can lead to loss of critical geometric information. This sparse and incomplete data directly impacts the reconstruction of the point cloud’s overall spatial structure, as well as the extraction of global feature information and local detail information, significantly reducing the inspection accuracy of the bearing rings [4,5]. Therefore, completing sparse and incomplete bearing ring point cloud data not only restores critical geometric features that are obscured or missed during measurement (such as raceway curvature, chamfer dimensions, and seal groove parameters) but also enables the reconstruction of a complete three-dimensional surface topology through multi-scale feature fusion techniques [6].

Existing point cloud completion methods can be categorized into traditional approaches and deep-learning-based methods. Traditional methods primarily employ geometric rule-based approaches or template-matching techniques for completion. The former imposes certain requirements on the symmetry and regularity of the original point cloud and fails to achieve accurate completion results for complex shapes or point cloud data with significant local variations. The latter necessitates the construction of an exhaustive, complete model database, demands high precision in template selection and matching, and involves time-consuming matching and optimization processes [7,8,9]. In recent years, the rapid advancement of deep learning methods has established them as a crucial technical approach for point-cloud completion. Early point cloud completion efforts involved mapping point clouds onto voxel grids before feeding them into 3D convolutional networks for reconstruction. However, the inherently disordered and unstructured nature of 3D point clouds poses significant challenges for direct processing using neural networks. The introduction of PointNet [10] marked a milestone in point cloud deep learning. It directly processes unordered point clouds using a multilayer perceptron (MLP) and incorporates max pooling to preserve global features, thereby addressing the disorder inherent in point clouds. Subsequently, Yuan et al. [11] built upon this foundation to propose the Point Completion Network (PCN), which is the first deep learning framework specifically designed for point cloud completion. The PCN employs an encoder–decoder architecture: the encoder extracts global features from the point cloud based on the PointNet structure, while the decoder combines a predefined 2D grid with these global features using the FoldingNet [12] structure to reconstruct the complete point cloud. However, this approach neglects the extraction of local detail features, resulting in the generation of point clouds that lack fine-grained details. Achlioptas et al. [13] proposed L-GAN (latent-space GAN), which was the first GAN-based point cloud completion network. However, this network does not obtain features from the original point cloud but instead passes data through a pre-trained encoder before converting it back to a point cloud via the decoder. This approach results in a structural information loss and suboptimal completion performance. Huang et al. [14] introduced PF-Net, which enhances the point cloud generation module architecture. It employs a multiresolution encoder and a pyramid-structured decoder for the hierarchical prediction of missing point clouds, integrating multilevel loss functions and adversarial loss functions to optimize the training process.

With the widespread application of transformer models in fields such as natural language processing and computer vision, researchers have discovered that their attention mechanism possesses permutation invariance and strong feature-learning capabilities, making it highly suitable for point cloud processing and analysis. Yuan et al. [15] proposed PoinTr, which models point-cloud completion as a set-to-set prediction task. Input point clouds are abstracted into point proxies, and a geometry-aware transformer is introduced to encode both global and local features uniformly, enhancing the adaptability and accuracy of completion. Xiang et al. [16] introduced SnowflakeNet, incorporating a skip attention mechanism within the Snowflake Point Deconvolution (SPD) layer to capture local features. It employs a recursive snowflake-like generation strategy to progressively enrich local details while preserving global structural consistency. Lei et al. [17] proposed an attention-based tree point-cloud completion network. Employing an encoder–decoder architecture and integrating a Multi-functional Attention Feature Enhancement (MAFE) module, it effectively mitigates local density sensitivity issues and enhances recovery capabilities for sparse foliage structures and complex hierarchical point clouds.

Although deep learning-based point cloud completion methods have made significant progress, existing research has primarily focused on objects with distinct classification features in indoor or street scenes, such as aircraft, tables, chairs, and automobiles. Most of these studies utilized datasets, such as Shapenet and KITTI. Compared to the aforementioned research subjects, precision parts, such as bearing rings, possess more intricate and complex structures, placing higher demands on the performance of point cloud completion networks. Furthermore, current point cloud completion models predominantly emphasize global feature extraction while neglecting local features and neighborhood relationships. This approach struggles to accurately reconstruct locally missing structures in bearing ring point clouds, resulting in significant limitations.

To address these challenges, this study draws inspiration from transformer-based approaches and other point cloud completion studies to propose an attention-based network for precision point cloud completion. Within the point cloud input module, we introduce a Curvature-Weighted Farthest Point Sampling (CW-FPS) method that prioritizes sampling points in high-curvature regions to preserve geometric details. The encoder module incorporates spatial attention and multiscale feature extraction to reduce the sensitivity to local density and enhance multiscale representation capabilities. The decoder module performs hierarchical prediction of the missing data. The discriminator module employs an MLP classifier with a biased attention mechanism to improve sensitivity to geometric variations in point clouds. To evaluate the performance of the proposed network, a virtual bearing ring point cloud completion dataset (referred to as the Bearing Ring Completion Dataset) was constructed using the SolidWorks 3D modeling software (SolidWorks 2024). The experimental results on this dataset demonstrate that the proposed method outperforms other mainstream point cloud completion networks in bearing ring point cloud completion tasks.

## 2. Fundamental Principles of the Proposed Network

**Figure 1:**
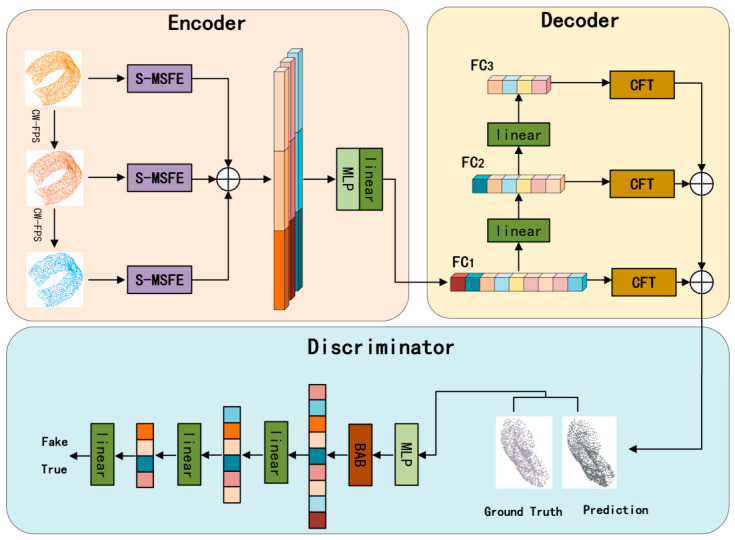
Network principle framework diagram of this study.

The framework of the proposed attention-enhanced feature-based point-cloud completion network for precision parts is illustrated in Figure 1. The overall architecture consists of a curvature-weighted sampling module, a multi-scale feature extraction module based on a spatial attention mechanism (S-MSFE), a multi-stage decoder, an adversarial generation module, and a loss function. In the encoder stage, the curvature-weighted sampling feature extraction module and spatial attention mechanism were introduced to extract both local and global features from the incomplete input point cloud, followed by multilevel feature fusion. The multiscale features extracted by the encoder are then passed to the decoder, which performs hierarchical and progressive prediction of the missing regions in the point cloud based on the encoded multilevel features. Finally, the adversarial generation module discriminates the refined predicted point cloud to produce a final complete and detailed reconstruction. The following sections provide detailed descriptions of each module.

### 2.1. Curvature-Weighted Sampling Module (CW-FPS)

**Figure 2:**
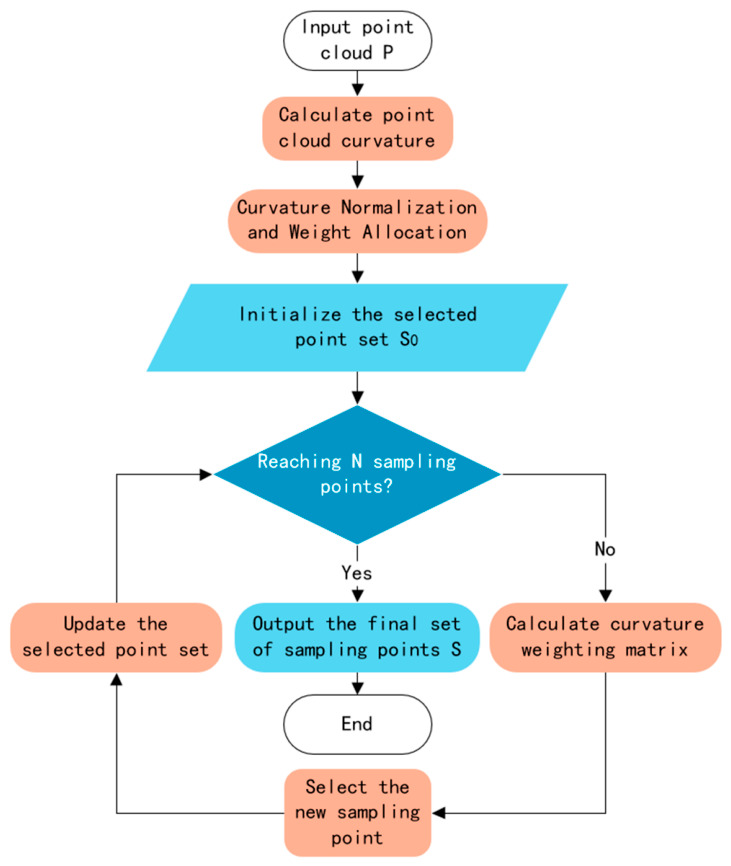
Flowchart of the CW-FPS Algorithm.

In the point cloud input module, traditional networks typically employ Farthest Point Sampling (FPS) to obtain three sets of raw point cloud data at different resolutions. However, FPS tends to lose local geometric features, making it difficult to meet the high-precision sampling requirements of precision parts. To address this issue, this study proposes a curvature-weighted farthest-point sampling (CW-FPS) method. A flowchart of this algorithm is shown in Figure 2. This method integrates the curvature information of each point and assigns corresponding weights, prioritizing sampling points in high-curvature regions (such as edges and corners). As a result, the CW-FPS can more effectively preserve the geometric details of the precision parts.

Specifically, the process involves the following steps:

(1) Curvature Calculation. First, for each point in the point cloud, the K-Nearest Neighbors (KNN) algorithm is used to determine its local neighborhood. The covariance matrix of the neighboring point set is then calculated as follows.

(1) $$C=\frac{1}{k}\sum_{j=1}^{k}(p_{j}-\bar{p}){\left(p_{j}-\bar{p}\right)}^{T}$$

where k is the number of points within the neighborhood. This article sets the number of nearest neighbors k = 30 in the experiment, and $\bar{p}=\frac{1}{k}\sum_{j=1}^{k}p_{j}$ represents the centroid of the neighborhood points. To enhance the sensitivity of curvature to subtle geometric changes, this paper introduces a curvature weight factor α = 0.8 when constructing KNN graphs, which is used to adjust the relative importance of distance and curvature in nearest neighbor selection. This value is determined through grid search on the validation set and can effectively improve feature discrimination.

The covariance matrix C is then decomposed into eigenvalues, yielding three eigenvalues. For $\lambda_{0}\leq \lambda_{1}\leq \lambda_{2}$, the curvature $\sigma_{i}$ of $p_{i}$ is given by:

(2) $$\sigma_{i}=\frac{\lambda_{0}}{\lambda_{0}+\lambda_{1}+\lambda_{2}}$$

(2) Curvature Normalization and Weight Assignment. The calculated curvature was then normalized into a weight $w_{i}$, scaled to the range [0,1]

(3) $$w_{i}=\frac{\sigma_{i}-\sigma_{\min}}{\sigma_{\max}-\sigma_{\min}}$$

where $\sigma_{\max}$ and $\sigma_{\min}$ are the maximum and minimum curvature values, respectively, within the entire neighborhood point cloud.

(3) Initialise the selected point set. Point $P_{0}$, which is farthest from the centroid of the point cloud, is chosen as the initial sampling point set $S_{0}$.
(4) Iterative sampling. First, we initialize a distance array D, where D[i] stores the minimum Euclidean distance from the point $p_{i}$ to the current sampled set S.

(4) $$D[i]=\min\left\{d(p_{i},S)\right\}$$

where $\left\{d(p_{i},S)\right\}$ represents the Euclidean distance from the current point $p_{i}$ to all points in the sampled set S.

Next, calculate the weighted distance $d_{wi}$ for each point $p_{i}$.

(5) $$d_{wi}=D[i]\times (1+\alpha \cdot w_{i})$$

where α is the curvature weighting factor that controls the degree of influence of the curvature on the sampling process.

Subsequently, the point $p_{new}$ with the maximum weighted distance $d_{wi}$ is selected and added to the sampled set S, and the sampled set S is updated.

(5) Termination conditions. Determine whether the number of sampled points in set S, has reached the target number N. If so, output the final sampled point set S; otherwise, repeat Step (4) until the number of sampled points in S reaches the target N, and outputs the final sampled point set S.

**Figure 3:**
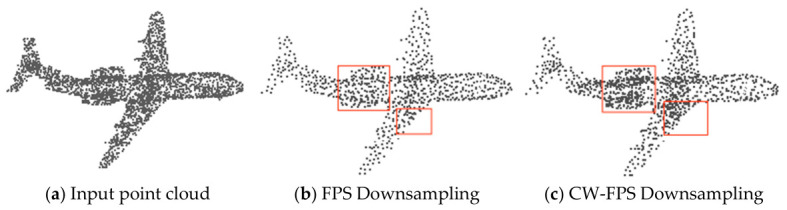
Comparison of Downsampling Effects Between CW-FPS Algorithm and FPS Algorithm.

Figure 3 shows a comparison between the downsampling effects of the traditional FPS method and the proposed CW-FPS algorithm on an airplane model from the ShapeNet-Part dataset. Compared with traditional FPS sampling, the proposed CW-FPS algorithm samples more points in key regions such as the fuselage and wings, resulting in a clearer overall contour. By incorporating local curvature information into the distance metric of traditional farthest point sampling in a weighted form, the method maintains a relatively uniform spatial distribution of the point cloud, while adaptively adjusting the sampling density of different feature regions according to the curvature (local geometric complexity). This achieves an evolution from “uniform sampling” to “feature-sensitive sampling.”

### 2.2. Multi-Scale Feature Extraction Module Based on Spatial Attention Mechanism (S-MSFE)

**Figure 4:**
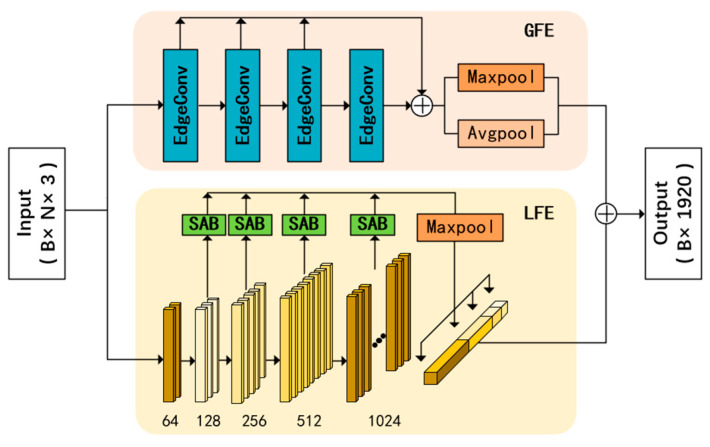
Structure of the Multi-Scale Feature Extraction Module.

The function of the feature extractor is to extract both local and global features from the incomplete point cloud input and map them into shape encoding. This module comprises two components: a Local Feature Extraction (LFE) unit and a Global Feature Extraction (GFE) unit. The complete structure of the multiscale feature-extraction module is shown in Figure 4.

For global features, this study draws inspiration from the dynamic graph convolution (EdgeConv) concept in the DGCNN. The detailed structure is shown in Figure 4. At each layer, the algorithm recalculates the neighborhood relationships between points based on the high-dimensional feature output from the previous layer.

Specifically, if the output of the layer is represented as $X^{l}$, then an N-nearest neighbor graph $G^{l}=(V,\theta )$ is constructed in the feature space using the k−NN algorithm. The vertex set V represents the points in the point cloud, whereas the edge set θ consists of directed edges. For example, the edge from point $x_{i}$ to its k nearest neighbors is denoted as $\left(i,j_{1}),(i,j_{2}),\ldots ,(i,j_{k}\right)$. For each directed edge (i,j), the edge feature is defined as $e_{ij}=h_{\Theta }(x_{i},x_{j}-x_{i})$, where $h_{\Theta }$ is a nonlinear function parameterized by the learnable parameter. Subsequently, for each point, the edge features of all the associated edges are aggregated channel-wise using an aggregation function, yielding a new feature representation for that point: $x_{i}^{\prime }=\max_{j:(i,j)\in \theta }e_{ij}$. Subsequently, the multilevel edge features obtained through the four layers of edge convolutions were concatenated. Global max pooling (MaxPool) and global average pooling (AvgPool) were then applied to these concatenated features. The outputs from both pooling operations were concatenated to form the final global feature vector.

**Figure 5:**
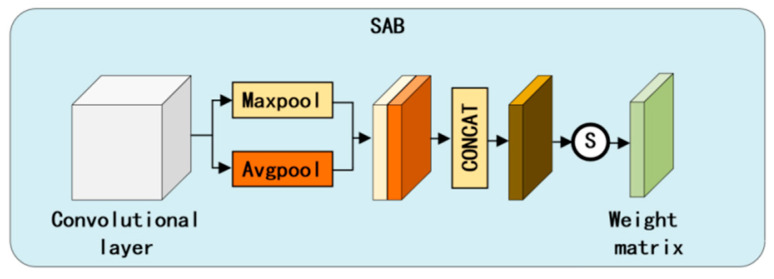
Structure of the Spatial Attention Block (SAB).

For local features, this study draws on the CMLP module from PF-Net and introduces a Spatial Attention Block (SAB) to apply spatial attention weighting to the features at each layer, thereby enhancing key features. Figure 5 illustrates the schematic of the spatial attention mechanism. Although point clouds are disordered, they can be transformed into structured feature representations by constructing local neighborhoods such as K-nearest neighbors. The spatial attention mechanism (SAB) in this network acts on the structured feature map extracted by hierarchical convolution. SAB aggregates the responses of different channels to the same spatial position (corresponding to local regions in the point cloud) by performing max pooling and average pooling on feature maps in the channel dimension, generating attention weights that reflect the importance of each local region. This enables the network to adaptively enhance its focus on high curvature structures such as edges and channels, while suppressing flat or noisy areas. Therefore, SAB essentially recalibrates the features that represent three-dimensional local geometric relationships in the feature space through learned spatial weights, effectively capturing the structural information that is crucial for point cloud completion. By combining the structured convolutions of the CMLP with the spatial attention mechanism, this method significantly improves the ability of the model to perceive local geometric structures and extract critical features.

First, a series of two-dimensional convolutional layers (Conv2d) and batch normalization layers (BatchNorm2d) were used to extract the convolutional features at different levels, encoding each point into five levels: 64, 128, 256, 512, and 1024. For the feature map output from a given convolutional layer, both max-pooling and average-pooling operations are performed. Subsequently, the results from these two pooling operations are concatenated along the channel dimension to form a new tensor that fuses the two distinct statistical features. A convolutional layer is then applied to this concatenated feature, reducing the number of channels to one. A sigmoid activation function is then applied to generate a spatial attention matrix, where each element’s value between 0 and 1 represents the weight assigned to the corresponding spatial location in the original feature map. This spatial attention matrix was multiplied element-wise with the original input feature map, thereby enhancing the key features of the local geometric structure. Finally, following the CMLP processing philosophy, the last four layers underwent max pooling to obtain multidimensional feature vectors $f_{i}:=128,256,512,1024$, where i=1,…,4. The pooled features were then concatenated to form a combined latent feature vector of dimension 1920. This latent feature vector, containing both high- and low-level features, serves as the final local feature vector.

After extracting local and global features at different scales through the LFE and GFE units, respectively, the features are added together and then passed through a fully connected layer to obtain the final feature vector.

In the multi-scale feature extraction module based on a spatial attention mechanism (S-MSFE), each module first performs feature fusion and dimensionality reduction within the module after independently extracting local features (LFE) and global features (GFE). Specifically, the local feature extraction unit outputs a 1920-dimensional combined feature vector, while the global feature extraction unit outputs a 512-dimensional feature vector; The two are concatenated in the channel dimension to form a 2432-dimensional intermediate fusion feature. Subsequently, the feature undergoes nonlinear transformation and compression through a fully connected layer (with an output dimension of 64), extracting the rich information of each S-MSFE module into a compact 64-dimensional representation. Three parallel S-MSFE modules in the encoder each output a 64-dimensional feature vector. Next, feature fusion is performed between modules: these three 64-dimensional vectors are concatenated in the feature dimension to form a 192-dimensional fused feature that aggregates information from different scales and receptive fields. Finally, these 192-dimensional features are fed into a multi-layer perceptron (MLP) for final deep integration and dimension regularization. The MLP structure is “input (192), connected layer (512, ReLU), fully connected layer (256, ReLU) and output (256)”, ultimately generating a 256-dimensional global feature encoding, which is passed to the decoder as the output of the encoder.

### 2.3. Multi-Stage Decoder

Traditional decoders typically generate point clouds directly through fully connected layers or deconvolution networks. However, this approach often fails to fully leverage the feature information extracted by the encoder. The decoder component in this study draws inspiration from the pyramid-based decoder concept of PF-Net, adopting a multistage decoding method. It first produces a coarse and sparse point set, and then progressively refines these point sets to more accurately complete the incomplete point cloud.

**Figure 6:**
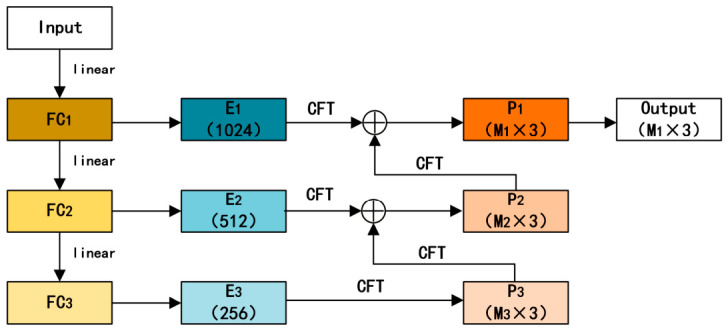
Architecture of the Cross-Stage Decoder.

Specifically, we first defined three fully connected layers with distinct output dimensions: ${FC}_{1}$, ${FC}_{2}$, and ${FC}_{3}$, with output dimensions of 1024, 512, and 256, respectively. Then, the final feature vector extracted by the encoder is successively passed through these three fully connected layers for dimensionality reduction, yielding three sub-feature vectors, $E_{1}$, $E_{2}$, and $E_{3}$, with feature dimensions of 1024, 512, and 256, respectively. These were used for phased point-cloud generation. During decoding, the network employs a multistage generation strategy, from coarse to fine. This process begins with the deepest subfeature $E_{3}$, which predicts an initial coarse point cloud $P_{3}\in {\mathbb{R}}^{M_{3}\times 3}$ through a fully connected layer. This serves as the skeleton for completing the point cloud, where $M_{3}$ denotes the number of points output by the layer. Subsequently, the sub-feature uses each point in the $E_{2}$ as the center point for the relative coordinate calculations. Through operations including convolution, feature concatenation, and tensor reconstruction, a denser medium-resolution point cloud $P_{2}\in {\mathbb{R}}^{M_{2}\times 3}$. Similarly, sub-feature $E_{1}$ utilizes a medium-resolution point cloud $P_{2}$ to generate the final point cloud $P_{1}\in {\mathbb{R}}^{M_{1}\times 3}$. The entire workflow is illustrated in Figure 6 below, where “linear” denotes the fully connected layer, and CFT encompasses operations including convolution, feature concatenation, and tensor reconstruction.

### 2.4. Adversarial Generative Module

**Figure 7:**
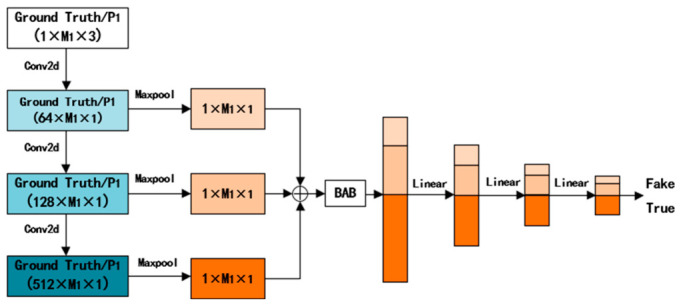
Structure of the Discriminator Module.

In the structure of a Generative Adversarial Network (GAN), there are generally two subnetworks: a generator and a discriminator. The preceding encoding and decoding processes constitute a generator. The discriminator in this study is designed based on the GAN framework while incorporating a Biased Attention Block (BAB) that introduces a bias matrix derived from geometric information. This enhances the sensitivity of the discriminator to geometric structural differences in point clouds, thereby improving its overall performance. The complete structure of the discriminator module is shown in Figure 7, where linear denotes a fully connected layer.

First, the final point cloud generated by the decoder and the ground truth point cloud were simultaneously input into the discriminator, where their depth features were extracted at multiple scales. Subsequently, these features underwent sequential weighted adjustments using BAB. Specifically, for each point of $p_{i}$, its neighborhood was selected using K-Nearest Neighbors (KNN). The relative positions $\Delta p_{ij}=p_{i}-p_{j}$ between $p_{i}$ and other points within the neighborhood are then computed. These relative positions were mapped through a multilayer perceptron to yield the corresponding geometric biases. Based on these geometric biases, a biased attention weight matrix is calculated. For the query vector $Q_{i}$ and key vector $K_{j}$, the biased attention weight is $\alpha_{ij}=\frac{Q_{i}\cdot K_{j}}{\sqrt{d_{k}}}+b_{ij}$, where $d_{k}$ is the feature dimension of the key vector used to scale the dot product result to stabilize gradients. Next, the attention scores were normalization $w_{ij}=\frac{\exp\left(\alpha_{ij}\right)}{\sum_{l\in N(i)}\exp\left(\alpha_{il}\right)}$, and the normalized attention weights were applied to weight the sum of the value vector $V_{j}$, yielding the modified feature $o_{i}=\sum_{j\in N(i)}w_{ij}V_{j}$.

The multiscale features refined by the BAB module are concatenated along the channel dimension to form a fused feature representation. The multi-scale features corrected by the BAB module are concatenated in the channel dimension to form a fused feature representation. The core innovation of the BAB module lies in the geometric bias obtained by calculating the local geometric relationships of points through relative coordinates, which is then explicitly injected into the calculation of attention weights. The traditional self-attention mechanism mainly relies on feature similarity, while the geometric bias term introduced by BAB modulates the attention weights simultaneously by feature similarity and local spatial structure similarity. For areas with unreasonable or defective structures in the generated point cloud, there will be significant differences in the geometric relationship between the generated point cloud and the real point cloud in the corresponding local area, resulting in abnormal changes in the attention weights at that location. This design greatly enhances the sensitivity of the discriminator to subtle geometric differences between generated point clouds and real point clouds, enabling it to distinguish authenticity not only from overall features but also from local structural consistency.

Finally, the fused features undergo nonlinear transformation and dimension reduction through multiple fully connected layers, outputting a one-dimensional scalar that represents a binary classification result indicating whether the input point cloud is “real” or “generated.”

### 2.5. Loss Function

This study employs the Chamfer Distance (CD) as the loss function of the network. The Chamfer Distance measures the similarity between two point clouds by calculating the sum of the average minimum distances from each point in a point cloud to its nearest point on the other. It quantifies the difference between the predicted and ground-truth point clouds, and by minimizing this difference, the model’s ability to fit the data is progressively improved.

Assuming that A represents the point cloud completed by the network and B is the corresponding ground truth point cloud, with x and y denoting any points in A and B, respectively, the calculation formula for CD is as follows.

(6) $$CD(A,B)=\frac{1}{\left|A\right|}\sum \min_{x\in A,\;y\in B}{∥x-y∥}_{2}^{2}+\frac{1}{\left|B\right|}\sum \min_{x\in A,\;y\in B}{∥y-x∥}_{2}^{2}$$

A smaller chamber distance indicates better completion performance for the missing parts, whereas a larger value suggests poorer completion quality. Because the decoder used in this study produces multi-stage outputs, the Chamfer Distances between the completed point clouds at three different resolutions and their corresponding ground truth point clouds are weighted and summed. Therefore, the Chamfer loss function of the proposed network, denoted as $L_{com}$, is defined as follows.

(7) $$L_{com}=CD\left(P_{1},Y_{gt1}\right)+2\varepsilon CD\left(P_{2},Y_{gt2}\right)+\varepsilon CD\left(P_{3},Y_{gt3}\right)$$

where $CD\left(P_{1},Y_{gt1}\right)$, $CD\left(P_{2},Y_{gt2}\right)$, and $CD\left(P_{3},Y_{gt3}\right)$ represent the fine point $P_{1}$, sub-fine point $P_{2}$, and skeletal point $P_{3}$, respectively, along with their corresponding ground truth point clouds $Y_{gt1}$, $Y_{gt2}$, and $Y_{gt3}$; ε denotes the weighting coefficient (ε<1), respectively.

In the adversarial generation module, the discriminator computes the loss between its output and corresponding labels to determine the authenticity of the generated results. Therefore, the primary purpose of introducing the adversarial loss function is to minimize the generator’s loss, encouraging it to produce samples that increasingly resemble real samples, thereby enhancing the discriminator’s ability to distinguish between real and generated samples.

(8) $$L_{adv}=\sum_{1\leq i\leq S}\log(D(y_{i}))+\sum_{1\leq j\leq S}\log(1-D(F(x_{i})))$$

where D denotes the discriminator’s output, with the inputs being the true point $y_{i}$ of the missing portion and the predicted completion value $F(x_{i})$ for a point $x_{i}$ in the point cloud to be completed. S represents the size of the point cloud.

The Chamfer loss function and the adversarial loss function together form a joint loss function that is used to optimize the model parameters collaboratively:

(9) $$L=\lambda L_{com}+(1-\lambda )L_{adv}$$

where $L_{com}$ denotes the Chamfer Distance (CD) loss function, $L_{adv}$ represents the adversarial loss function, and λ represents the loss function weighting coefficient.

## 3. Experiments and Results Analysis

### 3.1. Construction of the Experimental Dataset

Although numerous public datasets are available, there is a lack of datasets specifically designed for the point cloud completion of mechanical components, such as bearing rings. Drawing inspiration from Huang Shichang [9], who constructed a gear component dataset using 3D modeling software, this study built a dataset for bearing ring completion.

**Figure 8:**
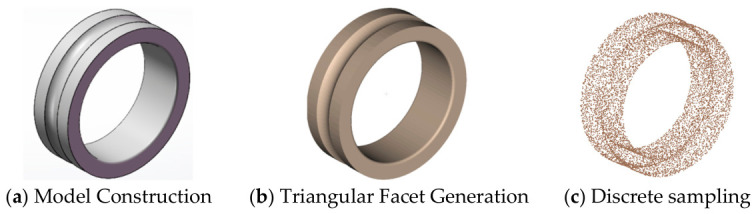
Construction Process of the Bearing Ring Dataset.

First, SolidWorks software was used to generate CAD models of various types of bearing rings, including deep groove ball bearings, angular contact ball bearings, cylindrical roller bearings, and double-row cylindrical roller bearings. These models were then saved in the commonly used STL format, serving as input files for subsequent point cloud discretization. When extracting point clouds from the STL models, 3D data were derived from the vertex coordinates of each triangular facet. By adjusting the resolution settings in SolidWorks, triangular facets of different sizes and levels of detail can be obtained. Next, 3D point cloud processing software, such as MeshLab (This article uses clodcompare2023.12) or CloudCompare (This article uses clodcompareV2.13.2), was employed to extract the vertex coordinates of these triangular facets, thereby obtaining the 3D coordinate data of the facet vertices. Through this approach, the point clouds generated at high resolution were dense and evenly distributed, effectively capturing the surface geometry of the object. The specific process is illustrated in Figure 8.

In total, 1520 models of different types of bearing inner and outer rings were generated in this study, including 500 deep groove ball bearings, 400 angular contact ball bearings, 320 cylindrical roller bearings, and 300 double-row cylindrical roller bearings. These models were discretized and sampled at various resolutions, resulting in 2120 point-cloud datasets with different densities but uniform distributions. The datasets were then divided into training, testing, and validation sets in a ratio of 8:1:1.

The bearing ring completion dataset constructed in this article is derived from an idealized CAD model, aiming to provide a controllable and accurate preliminary validation basis for the proposed network. Although there is a gap between the point cloud obtained by real 3D scanners and those affected by noise, specular reflection, and complex occlusion, this synthesized data can effectively evaluate the core ability of the network in restoring known geometric structures. To simulate data loss caused by occlusion or measurement limitations in actual scanning, this paper uses a combination of random cropping and viewpoint occlusion to generate incomplete input point clouds. Firstly, one or more continuous 3D holes are randomly generated on the surface of the complete point cloud (random cropping) to simulate large-scale loss caused by severe occlusion; Secondly, by simulating the areas that are not visible to the scanner from a specific perspective (viewpoint occlusion), realistic local incompleteness is generated. This comprehensive simulation strategy helps network learning to robustly complete multiple missing patterns. Future work will focus on introducing real industrial scanning data to further validate and optimize the performance of networks under more complex real-world conditions.

### 3.2. Experimental Environment

The experiments in this study were conducted in an Ubuntu 22.04 environment using the PyTorch 2.3.0 framework. The hardware configuration was as follows: the CPU was an Intel(R) Xeon(R) E5-2699 v4 with 128 GB of memory, and the GPU was an NVIDIA GeForce RTX 3090 SUPER with 24 GB of VRAM. The main programming languages used were Python 3.11 and CUDA 12.1. Based on the local computing environment, the number of training iterations (epochs) was set to 200, and the batch size was set to 32. The initial learning rate was set to 0.0002 with a weight decay coefficient of 0.001. The Adam optimizer was employed to optimize the network training process. In terms of network input and output, the original point cloud input by the encoder is fixed at 8192 points, and the number of clipping points is fixed at 2048 points. The complete point cloud output by the decoder and the ground truth point cloud used for loss calculation both contain 8192 points.

### 3.3. Evaluation Indicators

Referring to the evaluation metrics commonly used in existing point cloud completion methods on public datasets, this study adopts the following three metrics for testing: the Chamfer Distance (CD) error introduced in Section 2.5, the Pred → GT (Prediction-to-Ground Truth) error, and GT → Pred (Ground Truth-to-Prediction) error proposed by Lin C [18]. The calculation methods for the Pred → GT and GT → Pred errors are as follows.

(10) $$\left\{ \begin{array}{c} \mathrm{Pr}\;ed\to GT=\min_{p_{j}\in T}{∥{\hat{p}}_{i}-p_{j}∥}_{2}\\ GT\to \mathrm{Pr}\;ed=\min_{{\hat{p}}_{i}\in P}{∥p_{j}-{\hat{p}}_{i}∥}_{2} \end{array}\right.$$

In the equation, Pred → GT represents the distance from a point ${\hat{p}}_{i}$ in the predicted point cloud to its nearest point in the ground truth point cloud, whereas GT → Pred represents the distance from a point $p_{j}$ in the ground truth point cloud to its nearest point in the predicted point cloud. Here, P and T denote the predicted point cloud and ground-truth point cloud, respectively. The Pred → GT error measures the difference between the predicted results and ground truth by calculating the average squared distance from each point in the predicted point cloud to its nearest point in the ground truth point cloud. The GT → Pred error indicates the extent to which the ground-truth point cloud is covered by the predicted shape, computed as the average squared distance from each point in the ground-truth point cloud to its nearest point in the predicted point cloud.

### 3.4. Comparative Experiments

#### 3.4.1. Comparative Experiments Based on Existing Public Datasets

This study uses the ShapeNet-Part dataset, employed in PF-Net, as the benchmark dataset for comparing various state-of-the-art point cloud completion methods. The mainstream networks proposed in recent years—PCN, L-GAN, and PF-Net—were selected as baselines. All the methods were tested under the same experimental environment as the proposed method. The test set was input into the trained point cloud completion networks, and the CD, Pred → GT, and GT → Pred errors were calculated, as shown in Table 1 and Table 2. It can be observed that for the given completion objects, the proposed network achieved the lowest errors across all three metrics in the Airplane, Bag, Car, Chair, and Guitar categories. In the Cap category, the GT → Pred error was slightly higher than that of the second-best PF-Net, but the CD error and Pred → GT error reached optimal values. Compared with PF-Net, which achieved the best results in quantitative completion experiments, the proposed network reduced the CD, Pred → GT, and GT → Pred errors by 10.8%, 4.7%, and 8.1%, respectively.

Table 3 presents a visual comparison of the completion effects for several networks. It can be seen from the table that the completion effect of the network in this study is the most complete, uniform, and smooth. The completion effects of the PCN and L-GAN were relatively poor and differed significantly from the real data. In addition, compared with the PF-Net point cloud completion network, the completion effect of the network in this study is closer to real data. Specifically, for airplanes, PF-Net failed to restore the shape of the airplane head during completion, with fewer generated points in the head area and a failure to accurately restore the shape of the real airplane head; more discrete points were generated. For Bag, the completion effect of the network in this study is more complete and uniform than that of PF-Net. For Cap, although the completion effect of the network in this study is not as uniform as that of PF-Net, the network in this study has clearer contours and better boundary point completion effects. For the Car and Chair, the completion effect of the network in this study is more uniform and closer to the true point cloud than that of PF-Net. For Guitar, the completion effect of the network in this study has clearer contours and better boundary point completion effects compared to PF-Net. Overall, the network in this study has high completion performance and robustness for fine point clouds.

#### 3.4.2. Comparison Experiments Based on the Proposed Dataset

The previous section (Section 3.4.1) experimentally verified the effectiveness and superiority of the proposed method on the benchmark dataset, ShapeNet-Part. This section validates the completion performance of the proposed network by using the bearing ring completion dataset constructed in this study.

The proposed network was quantitatively compared with the PF-Net point cloud completion network in terms of the Pred → GT error, GT → Pred error, and CD error. The experimental results are presented in Table 4 and Table 5, respectively. It can be seen that, compared with PF-Net, the proposed network achieved the lowest error values in the completion of deep groove ball bearings, angular contact ball bearings, and double row cylindrical roller bearings. Although the CD error of the proposed network in the completion of the cylindrical roller bearings was slightly higher than that of PF-Net, the average error across all categories was smaller. Compared with the PF-Net point-cloud completion network, the Pred → GT, GT → Pred, and CD errors were reduced by 16.3%, 16.2%, and 9.3%, respectively. This indicates that in the completion task of fine point clouds with richer geometric features, the proposed network, by applying curvature-weighted sampling instead of farthest point downsampling, obtained more delicate geometric information. Meanwhile, the introduction of the spatial attention mechanism and bias attention mechanism not only reduced the traditional network’s sensitivity to the local density of bearing ring point clouds but also enhanced the network’s sensitivity to the geometric structure differences in point clouds, thereby improving the completion performance of the network to a certain extent.

Compared with PF-Net, the proposed network achieved significant improvements in the completeness, uniformity, and authenticity of the completion and was closer to the real point cloud. In particular, in areas with significant feature changes, the proposed network demonstrates a more delicate completion effect. The specific completion effects are presented in Table 6. This also proves the effectiveness of the curvature-weighted sampling module and attention mechanism introduced in the proposed network.

Experimental tests have shown that the network proposed in this paper exhibits good robustness to both random sparse loss and continuous block loss caused by severe occlusion, further verifying its applicability in practical complex measurement scenarios.

#### 3.4.3. Statistical Analysis Experiment

To evaluate the statistical significance of the performance difference between this paper’s network and mainstream point cloud completion networks, and to exclude the influence of random fluctuations, this paper conducted supplementary statistical analysis on the results of comparative experiments. Specifically, on the test sets of ShapeNet-Part and the self-built bearing ring dataset, run 5 independent model inference experiments for each category, record the CD error (or other evaluation indicators) of each experiment, and calculate its mean (Mean) and standard deviation (Std). The results are shown in Table 7. Subsequently, a paired sample *t*-test was used to pairwise compare the average performance of our network and each baseline network across all categories at a significance level of α = 0.05. The results are shown in Table 8. In the table, the data format is “mean ± standard deviation” (unit: 10^−3^); The “*p*-value” column displays the statistical test results of the performance difference between the network proposed in this article and the PF Net point cloud completion network.

The statistical test results show that the average CD error (4.663 ± 0.041, mean ± standard deviation) of our network on the ShapeNet Part dataset is significantly lower than that of PF Net (5.232 ± 0.037), and the *p*-value is less than 0.01, indicating high statistical significance. This statistical analysis confirms that the performance improvement observed by our method compared to existing mainstream methods is systematic, rather than random variability during the training process.

### 3.5. Analysis of Model Complexity and Efficiency

To evaluate the practical applicability of the proposed network in industrial testing scenarios, this section reports its objective efficiency indicators and compares them with mainstream PCN, L-GAN, and PF Net point cloud completion networks. The results are shown in Table 8. The experiment was conducted in the same software and hardware environment (see Section 3.2), using a single RTX 3090 GPU to perform forward inference on a single point cloud sample with batch size 1, and the average time was calculated.

As shown in Table 8, due to the introduction of the multi-branch S-MSFE module and attention mechanism, the number of parameters and computational complexity (FLOPs) of the network in this paper have increased compared to the baseline networks listed. The average inference time for a single point cloud is about 32.7 milliseconds (i.e., about 30 FPS). PCN and L-GAN, as earlier classical networks, have relatively simple structures and therefore have advantages in efficiency. Although the efficiency of this network is slightly lower than other networks, as shown in Section 3.4.1 and Section 3.4.2, it has achieved significant improvements in completion accuracy on various datasets. At a frame rate of 30 FPS, this network can still meet the real-time requirements of many industrial detection processes, achieving a more competitive balance between accuracy and efficiency.

### 3.6. Ablation Experiment

To verify the effectiveness of each part of the network in this article, the following ablation experiments were conducted: removing the spatial attention mechanism module (SAB) from the encoder to obtain network A; removing the bias attention mechanism (BAB) from the adversarial generation module to obtain network B; removing the curvature weighted sampling module (CW-FPS) to obtain network C; and using network D as the original network without changing any modules. Train the above networks under the same configuration and conduct completion experiments on the test set of the shapenet part dataset. The CD error results are shown in Table 9. It can be seen that the CD error of network A is the largest, followed by network B, and network C is the smallest, indicating that the SAB module is more helpful in extracting local and global features of point clouds. It contributes the most to the network, followed by the BAB module, and CW-FPS contributes the least to the network. In addition, the CD errors of network A, network B, and network C are all greater than that of network D, indicating that the SAB module, BAB module, and CW-FPS module have contributed to the overall completion effect of the network in this article.

## 4. Conclusions

This study proposes a precision part point cloud completion network based on attention-enhanced feature extraction and constructs a bearing ring completion dataset for training. The proposed network was built using an encoder–decoder architecture. In the encoder stage, a curvature-weighted sampling feature extraction module and a spatial attention mechanism are introduced to capture both the local and global features of the input incomplete point cloud, followed by multilevel feature fusion. The multilevel features extracted by the encoder are then fed into the decoder, which hierarchically and progressively predicts the missing regions of the point cloud. Finally, the adversarial generation module incorporates a biased attention mechanism to enhance the network’s sensitivity to geometric structural differences, enabling the discrimination of the generated point clouds and producing a complete and detailed reconstruction. Experimental results demonstrate that the proposed network achieves improved completion accuracy compared with existing mainstream completion methods on both the ShapeNet-Part and custom datasets developed in this study. Specifically, the average CD, Pred → GT, and GT → Pred errors are all lower than those of the PF-Net point cloud completion network, indicating enhanced completion authenticity. Moreover, the proposed network effectively completes the missing point clouds of various component types, verifying its robustness in point-cloud completion across different types of parts. The completed point cloud can effectively restore key geometric features such as bearing channels, providing a reliable data foundation for improving the accuracy of subsequent industrial tasks such as size measurement, and enhancing the engineering practicality of this method.

## Figures and Tables

**Table 1:** Comparison of CD on the ShapeNet-Part Dataset.

Category	CD (10^−3^)			
	PCN	L-GAN	PF-Net	This Study
Airplane	5.371	4.345	2.248	2.065
Bag	8.624	9.935	9.022	7.759
Cap	12.211	11.326	10.452	8.946
Car	4.836	5.541	4.496	4.306
Chair	4.443	4.678	4.173	3.959
Guitar	1.993	1.679	1.002	0.944
Average Value	6.246	6.251	5.232	4.663

**Table 2:** Comparison of Pred → GT/GT → Pred on the ShapeNet-Part Dataset.

Category	Pred → GT/GT → Pred (10^−3^)			
	PCN	L-GAN	PF-Net	This Study
Airplane	2.888/1.482	3.951/1.224	1.087/1.161	0.996/1.068
Bag	5.371/5.564	5.731/5.061	4.115/4.906	3.801/3.958
Cap	7.018/6.193	8.367/6.443	5.053/5.399	4.986/5.485
Car	3.576/2.259	4.265/2.792	2.587/2.039	2.446/1.861
Chair	2.221/2.217	3.360/2.428	2.171/2.002	2.081/1.877
Guitar	0.574/0.625	0.811/0.639	0.468/0.533	0.449/0.495
Average Value	3.608/3.057	4.414/3.098	2.580/2.673	2.459/2.457

**Table 3:** Comparison of Completion Performance on the ShapeNet-Part Dataset.

Category	Input	L-GAN	PCN	PF-Net	This Study	Ground Truth
Airplane	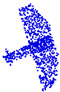	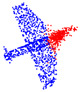	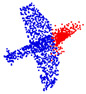	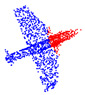	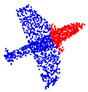	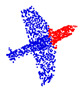
Bag	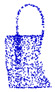	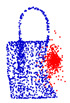	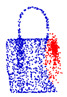	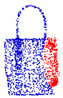	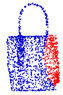	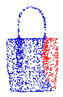
Cap	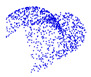	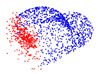	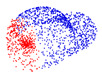	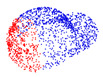	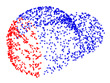	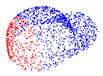
Car	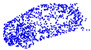	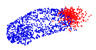	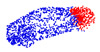	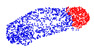	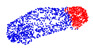	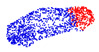
Chair	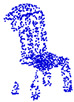	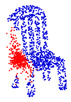	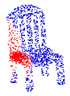	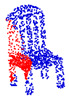	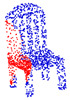	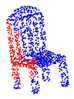
Guitar	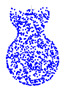	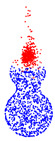	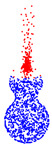	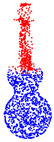	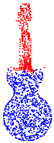	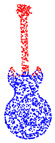

**Table 4:** Comparison of CD on the Bearing Ring Completion Dataset.

Category	CD (10^−3^)	
	PF-Net	This Study
Deep Groove Ball Bearings	0.615	0.536
Angular Contact Ball Bearings	0.469	0.364
Cylindrical Roller Bearings	0.589	0.610
Double Row Cylindrical Roller Bearings	0.517	0.482
Average Value	0.548	0.497

**Table 5:** Comparison of Pred → GT/GT → Pred on the Bearing Ring Completion Dataset.

Category	Pred → GT/GT → Pred (10^−3^)	
	PF-Net	This Study
Deep Groove Ball Bearings	1.471/0.856	1.252/0.716
Angular Contact Ball Bearings	1.013/0.545	0.836/0.472
Cylindrical Roller Bearings	1.390/0.802	1.121/0.644
Double Row Cylindrical Roller Bearings	1.213/0.670	1.048/0.567
Average Value	1.272/0.718	1.064/0.601

**Table 6:** Comparison of Completion Performance on the Bearing Ring Completion Dataset.

Category	Input	PF-Net	This Study	Ground Truth
Deep Groove Ball Bearings	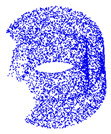	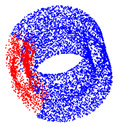	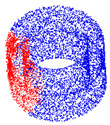	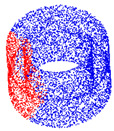
Angular Contact Ball Bearings	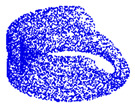	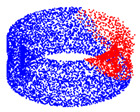	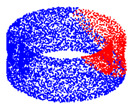	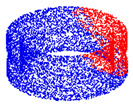
Cylindrical Roller Bearings	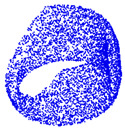	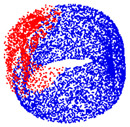	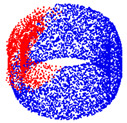	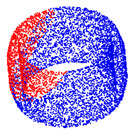
Double Row Cylindrical Roller Bearings	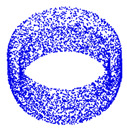	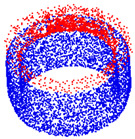	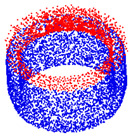	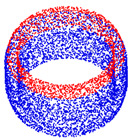

**Table 7:** Comparison of statistical analysis experiment of Different Models.

Category	PCN	L-GAN	PF-Net	Ours	*p*-Value
Airplane	5.371 ± 0.201	4.345 ± 0.183	2.248 ± 0.098	2.065 ± 0.045	<0.01
Bag	8.624 ± 0.315	9.935 ± 0.402	9.022 ± 0.287	7.759 ± 0.156	<0.01
Cap	12.211 ± 0.521	11.326 ± 0.487	10.452 ± 0.234	8.946 ± 0.189	<0.01
Car	4.836 ± 0.187	5.541 ± 0.221	4.496 ± 0.102	4.306 ± 0.091	<0.05
Chair	4.443 ± 0.176	4.678 ± 0.192	4.173 ± 0.121	3.959 ± 0.088	<0.05
Guitar	1.993 ± 0.085	1.679 ± 0.072	1.002 ± 0.041	0.944 ± 0.037	<0.05
Average	6.246 ± 0.132	6.251 ± 0.138	5.232 ± 0.037	4.663 ± 0.041	<0.01

**Table 8:** Comparison of Efficiency Indicators of Different Models.

Category	Parameter Count (Million)	FLOPs (G)	Average Inference Time (ms)
PCN	7.2	28.5	19.4
L-GAN	6.8	25.1	18.9
PF-Net	8.7	34.2	25.1
Ours	12.5	48.6	32.7

**Table 9:** Comparison of CD Errors in the Ablation Experiments on the ShapeNet-Part Dataset.

Model	SAB	BAB	CW-FPS	CD/10^3^
A		√	√	4.992
B	√		√	4.783
C	√	√		4.726
D	√	√	√	4.663

Note: √ indicates Include this module.

## Data Availability

The original contributions presented in this study are included in the article. Further inquiries can be directed to the corresponding author.
